# Internalization of CD239 highly expressed in breast cancer cells: a potential antigen for antibody-drug conjugates

**DOI:** 10.1038/s41598-018-24961-4

**Published:** 2018-04-26

**Authors:** Yamato Kikkawa, Yurie Enomoto-Okawa, Aiko Fujiyama, Takeshi Fukuhara, Nozomi Harashima, Yumika Sugawara, Yoichi Negishi, Fumihiko Katagiri, Kentaro Hozumi, Motoyoshi Nomizu, Yuji Ito

**Affiliations:** 1 Department of Clinical Biochemistry, Tokyo University of Pharmacy and Life Sciences, Tokyo, 192-0392 Japan; 2 Laboratory of Oncology, Tokyo University of Pharmacy and Life Sciences, Tokyo, 192-0392 Japan; 3 Department of Drug Delivery and Molecular Biopharmaceutics, Tokyo University of Pharmacy and Life Sciences, Tokyo, 192-0392 Japan; 4 Graduate School of Science and Engineering, Kagoshima University, Kagoshima, 890-0065 Japan; 5 Present Address: Department of Neurology, Graduate School of Medicine, Juntendo University, Bunkyo-ku, Tokyo, 113-8421 Japan

## Abstract

Antibody–drug conjugates (ADCs) are attractive in cancer therapy because they can directly bind to cancer cells and provide anticancer activity. To kill cancer cells with ADCs, the target antigens are required not only to be highly and/or selectively expressed on cancer cells but also internalized by the cells. CD239, also known as the Lutheran blood group glycoprotein (Lu) or basal cell adhesion molecule (B-CAM), is a specific receptor for laminin α5, a major component of basement membranes. Here, we show that CD239 is strongly expressed in a subset of breast cancer cells and internalized into the cells. We also produced a human single-chain variable fragment (scFv) specific to CD239 fused with human IgG_1_ Fc, called C7-Fc. The binding affinity of the C7-Fc antibody is similar to that of mouse monoclonal antibodies. Although the C7-Fc antibody alone does not influence cellular functions, when conjugated with a fragment of diphtheria toxin lacking the receptor-binding domain (fDT), it can selectively kill breast cancer cells. Interestingly, fDT-bound C7-Fc shows anticancer activity in CD239-highly positive SKBR3 cells, but not in weakly positive cells. Our results show that CD239 is a promising antigen for ADC-based breast cancer therapy.

## Introduction

Breast cancer is the most common cancer in female^1^. Many studies have attempted to identify the target molecules associated with breast cancer progression, to develop anticancer drugs. HER2, a member of the epidermal growth factor receptor family, which includes HER, EGFR, and ERBB, is well known as an antigen amplified in invasive breast cancer^2^. The pathogenic activity of HER2 in breast cancer makes it a good candidate for targeted antibody therapy. The humanized HER2 antibody trastuzumab (Herceptin) is currently approved for HER2-positive breast cancer treatment. However, because the overexpression of HER2 is observed in only 20% to 25% of breast cancer patients, the applicability of trastuzumab therapy is limited. Therefore, a novel target is needed for the diagnosis and treatment of HER2-negative breast cancer.

Chemotherapeutic drugs are frequently used for conventional cancer therapy. However, because the drugs usually show significant systemic toxicity, these approaches have narrow therapeutic indices. Therefore, new approaches are required to preferentially deliver chemotherapeutic drugs to cancer cells. Antibody–drug conjugates (ADCs) are cytotoxic drugs linked to target antigen-specific monoclonal antibodies (mAbs). They are able not only to maximize the efficacy of the cytotoxic drugs on cancer cells, but also to minimize exposure to normal cells. Thereby, ADCs are expected to improve therapeutic indices. Trastuzumab emtansine (T-DM1) is currently approved for a subset of patients that do not respond to trastuzumab-containing therapy^3^. T-DM1 combines trastuzumab and the potent antimicrotubule agent emtansine (DM1) using a unique linker. The cytotoxic mechanism is thought to involve T-DM1 bound to HER2 being internalized by receptor-mediated endocytosis, followed by the intracellular release of an active form of DM1, which in turn kills the cancer cells. T-DM1 is a successful ADC; however, because trastuzumab is used as the targeting antibody, this application is restricted to HER2-positive breast cancer patients. Therefore, novel antigens and targeting antibodies are required for the development of new ADCs.

CD239, also known as the Lutheran blood group glycoprotein (Lu) or basal cell adhesion molecule (B-CAM), is an Ig superfamily transmembrane protein. Lu was initially studied as the antigen of the Lutheran blood group system^4^, and B-CAM was identified as an up-regulated antigen in ovarian carcinoma^5^. Lu and B-CAM have the same extracellular domain, but different cytoplasmic tails. B-CAM lacks the COOH-terminal 40 amino acids of the Lu cytoplasmic tail. The Lu-specific cytoplasmic region carries an SH3-binding motif, a dileucine motif, and potential phosphorylation sites^6^. The common region of Lu and B-CAM cytoplasmic tails contains a spectrin-binding motif^7,8^. The cytoplasmic tails seem to be either differentially or similarly involved in intracellular signalling pathways. As described above, because the structure of B-CAM overlaps with that of Lu, it is difficult to distinguish between Lu and B-CAM within tissues. Hereafter, if Lu and B-CAM are not distinguished, they will be referred to as CD239.

The extracellular domain of CD239 contains one V-set, one C1-set, and three I-set domains (V-C1-I-I-I)^6,9,10^. CD239 specifically binds to laminin α5, a major component of basement membranes^11,12^. Therefore, CD239 is considered to contribute to cell adhesion to basement membranes. Laminin α5 assembles with β and γ chains to form heterotrimers found in many basement membranes in normal and diseased tissues. Our recent study showed that CD239 promotes the migration of lung carcinoma cells on laminin-511 (LM-511), which is composed of the α5, β1, and γ1 chains^13^. In addition, tumour cell migration on LM-511 is inhibited in the presence of a function-blocking antibody against CD239^13,14^. Several groups have shown that over-expression of CD239 is observed not only in ovarian carcinoma but also in skin cancer and hepatocellular carcinoma^15–17^. Hence, CD239 has been suggested as a useful antigen for diagnosis and development of antibody drugs.

In this study, we found that CD239 was strongly expressed in a subset of breast cancer tissues and cells. ADCs targeting CD239 showed anticancer effects in CD239-highly positive breast cancer cells. In addition, we produced a human scFv-Fc antibody against CD239 in mammalian cells and showed that it can serve as an antibody for the development of ADCs.

## Results

### Immunohistochemical analysis of CD239 in breast cancer

**Figure 1:**
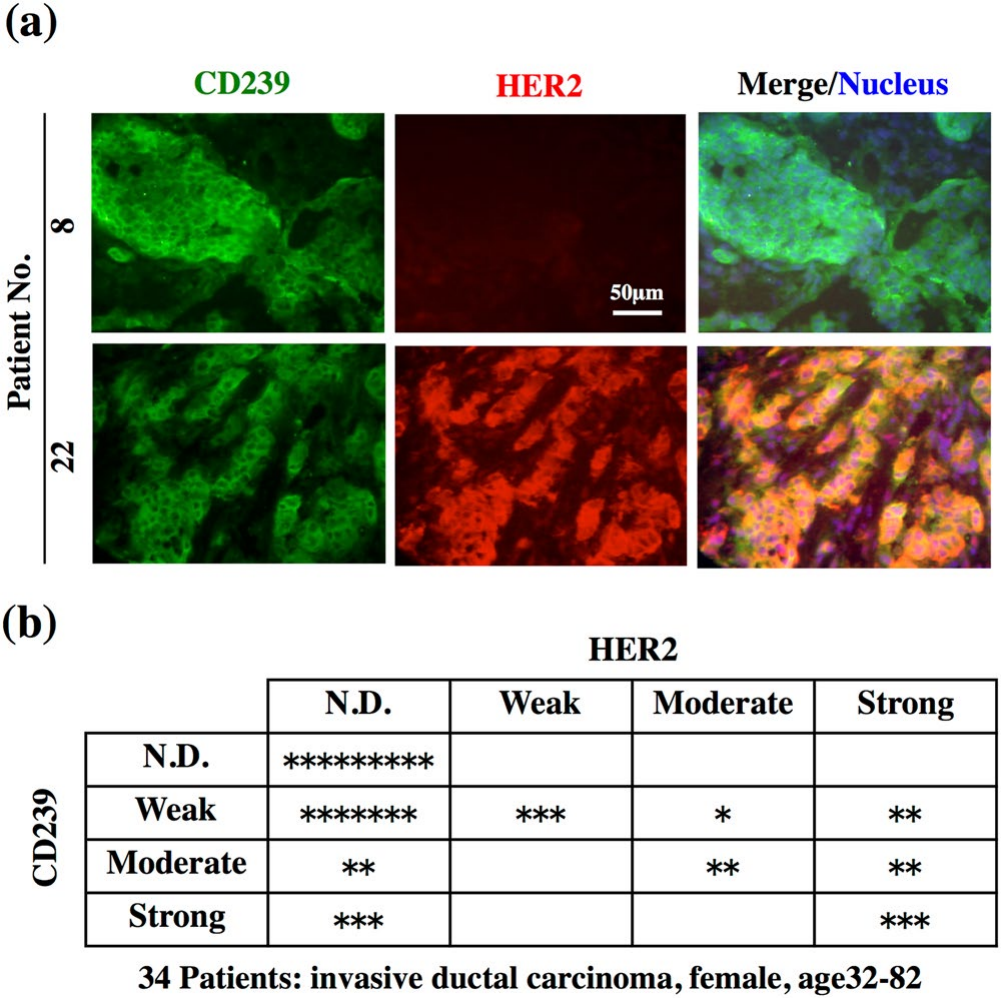
Expression of CD239 and HER2 in breast cancer tissues. (**a**) Frozen tissue sections were stained with antibodies against CD239 (green, left panels) and HER2 (red, centre panels). Merged images of CD239 and HER2 staining are shown in the right panels. Nuclei were counterstained with Hoechst 33258 (blue). CD239 is highly expressed in tissues of patients 8 and 22. (**b**) Summary of CD239 and HER2 expression in the breast cancer tissues tested. The scale of expression is described in Methods. N.D., not detected. *, a classified tissue.

We first determined the expression of CD239 in breast cancer tissues. The expression of CD239 was significantly increased in some invasive ductal carcinoma tissues (Fig. 1a). Of thirty-four patients tested, CD239 was positive in tissues of twenty-five patients (Table S1). CD239 was often localized on the entire surface of cancer cells in the highly positive tissues. In breast cancer, the expression of HER2 is usually tested to help choose the appropriate treatments. Our staining results for CD239 and HER2 in breast cancer tissues are summarized in Table S1. HER2 expression was usually accompanied by CD239 expression in breast cancer cells (Fig. 1b). On the other hand, strong CD239 expression was often observed in HER2-negative tissues. These results indicate that CD239 may serve as a molecular target for breast cancer therapy.

### Anticancer activity of fDT-bound mouse anti-CD239 mAbs

**Figure 2:**
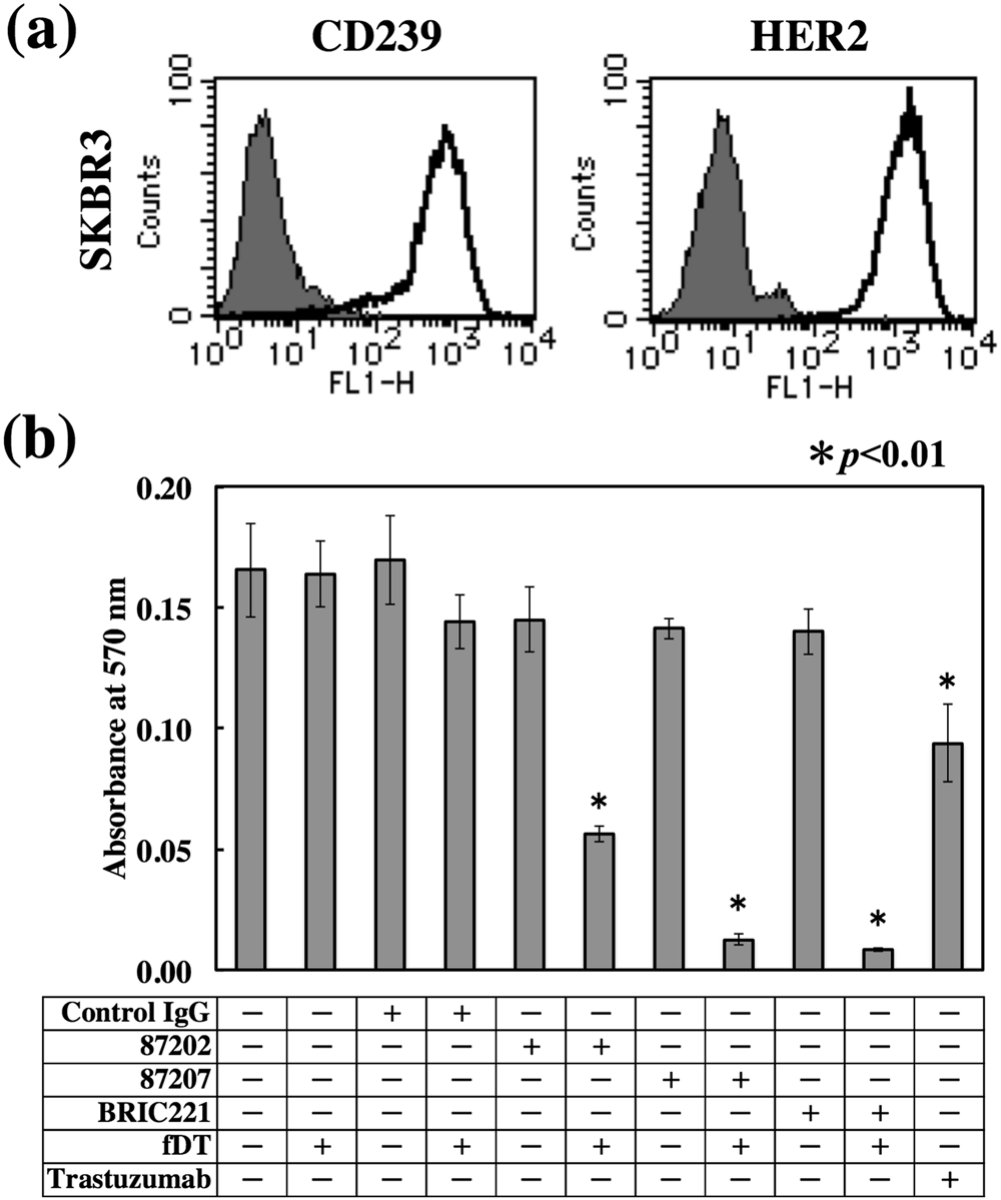
Cytotoxicity of fDT-bound anti-CD239 mouse mAbs on breast cancer cells. (**a**) Flow cytometric analysis of CD239 and HER2 expression in SKBR3 cells. As indicated in the histograms, the solid lines show anti-CD239 (left panel) and HER2 (right panel) mAbs. The grey fill indicates control IgG. SKBR3 cells highly expressed both markers. (**b**) Cytotoxicity of fDT-bound anti-CD239 mouse mAbs. The antibodies (87202, 87207, and BRIC221) were combined with a fragment of diphtheria toxin lacking the receptor-binding domain (fDT). SKBR3 cells were cultured in the presence of the antibodies alone or with bound fDT. Trastuzumab was used as a therapeutic antibody control. After 4 days’ culture, the number of cells was determined as described in Materials and Methods. fDT-bound antibodies suppressed proliferation of SKBR3 cells.

Flow cytometric analysis using anti-CD239 and -HER2 mAbs showed that SKBR3 cells strongly expressed not only HER2 but also CD239 (Fig. 2a). The results led us to examine whether anti-CD239 mAbs exhibited cytotoxicity towards SKBR3 cells as well as trastuzumab did. However, the antibody alone was not effective against CD239-highly positive SKBR3 cells (Fig. 2b). If CD239 is internalized into the tumour cell, the antibodies should be useful for ADCs. Recently, Yamaguchi *et al*. developed a sensitive screening method for selecting mAbs to be conjugated with anticancer drugs^18^. A fragment of diphtheria toxin fused with C1-3 domains of *Streptococcus* protein G (fDT) was linked to the antibodies. Three fDT-bound anti-CD239 mAbs suppressed proliferation of SKBR3 cells (Fig. 2b). Of these, mAb 87202, recognizing the N-terminal domain, was only moderately effective, suggesting that the internalization is dependent on the epitope of the antibody. These results indicate that CD239 is internalized by SKBR3 cells and serves as an antigen for ADCs.

### Production of human scFv-Fc antibody against CD239

**Figure 3:**
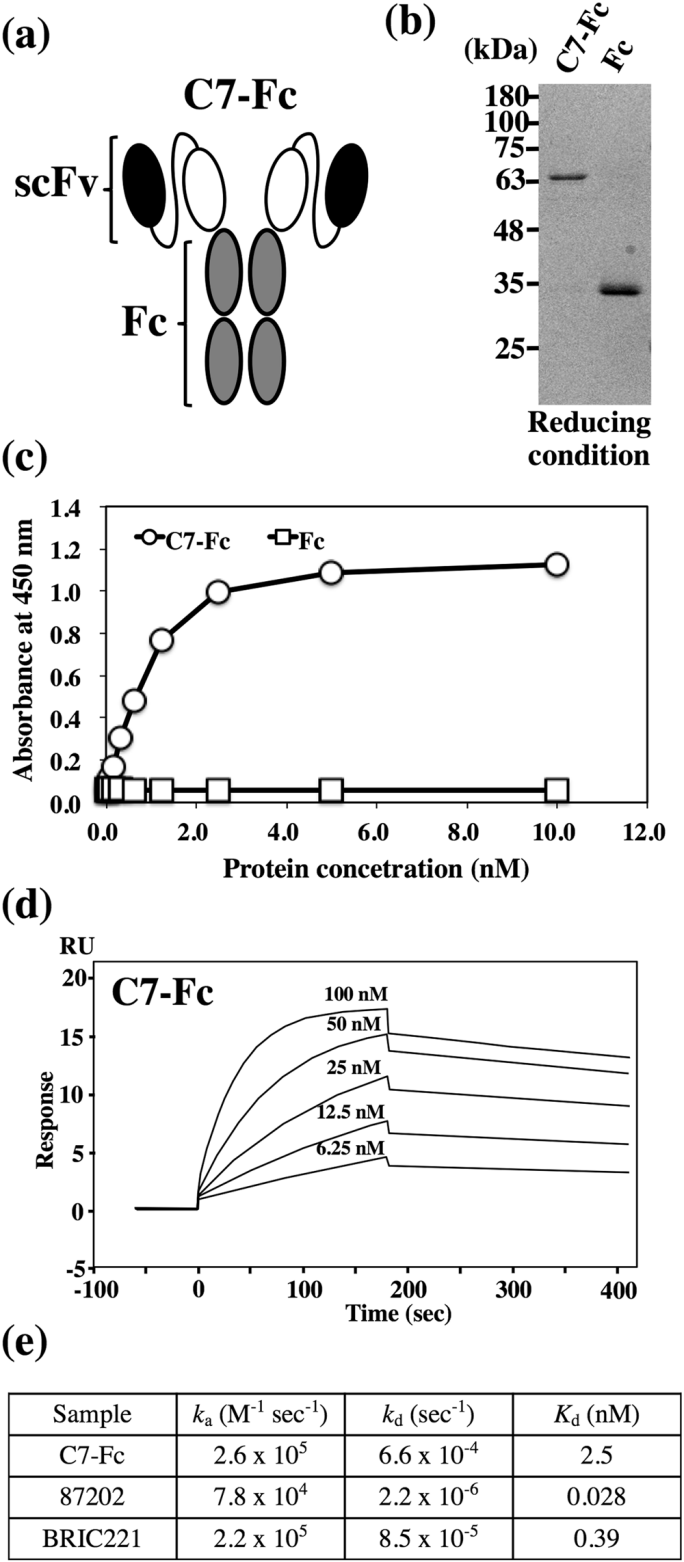
Production and characterization of C7-Fc. (**a**) Schematic representation of C7-Fc. scFv of the C7 phage was fused with human IgG_1_ Fc. The recombinant proteins were secreted from mammalian cells using the signal sequence of laminin γ2. (**b**) C7-Fc and Fc were purified from conditioned media using Protein A Sepharose. The proteins were separated on a 12.5% gel under reducing conditions. (**c**) ELISA using the C7-Fc antibody demonstrates binding to the recombinant extracellular domain of CD239 (Sol-Lu). (**d**) The binding affinity of C7-Fc was investigated using SPR. The sensorgrams represent the association and dissociation of the injected C7-Fc at different concentrations (as indicated) with and from (respectively) the immortalized recombinant CD239 (Lu-Fc). (**e**) Summary of kinetic parameters, *k*_a_ and *k*_d_ and the *K*_d_ of C7-Fc and mouse anti-CD239 mAbs.

We produced a human scFv-Fc antibody (C7-Fc) in which the scFv of the C7 phage clone was fused with human IgG_1_ Fc (Fig. 3a). The signal sequence of laminin γ2 was chosen to drive secretion of the C7-Fc antibody effectively from mammalian cells. SDS-PAGE analysis of purified C7-Fc revealed a single protein band corresponding to the expected molecular size (Fig. 3b). The binding activity of C7-Fc was confirmed using ELISA (Fig. 3c). C7-Fc bound to the recombinant extracellular domain of CD239 (Sol-Lu). We also measured the binding affinity of the C7-Fc antibody using surface plasmon resonance (SPR) (Fig. 3d). The kinetic parameters are summarized in Fig. 3e. The association rate constant (*k*_a_) of the C7-Fc antibody was 2.6 × 10^5^ M^−1^ sec^−1^ – moderate and similar to that of two mouse mAbs. On the other hand, the dissociation rate constant (*k*_d_) of the C7-Fc was 6.6 × 10^−4^ sec^−1^, which was larger than that of the two mouse mAbs, leading to a slow decrease of response (200 s) observed in the dissociation reaction. These results indicate that C7-Fc has a lower affinity (*K*_d_ = 2.5 nM) than the two mouse antibodies (*K*_d_ = 0.028 and 0.39 nM) but has sufficient affinity for binding to CD239.

### Anticancer activity of fDT-bound C7-Fc in CD239-highly positive breast cancer cell lines

**Figure 4:**
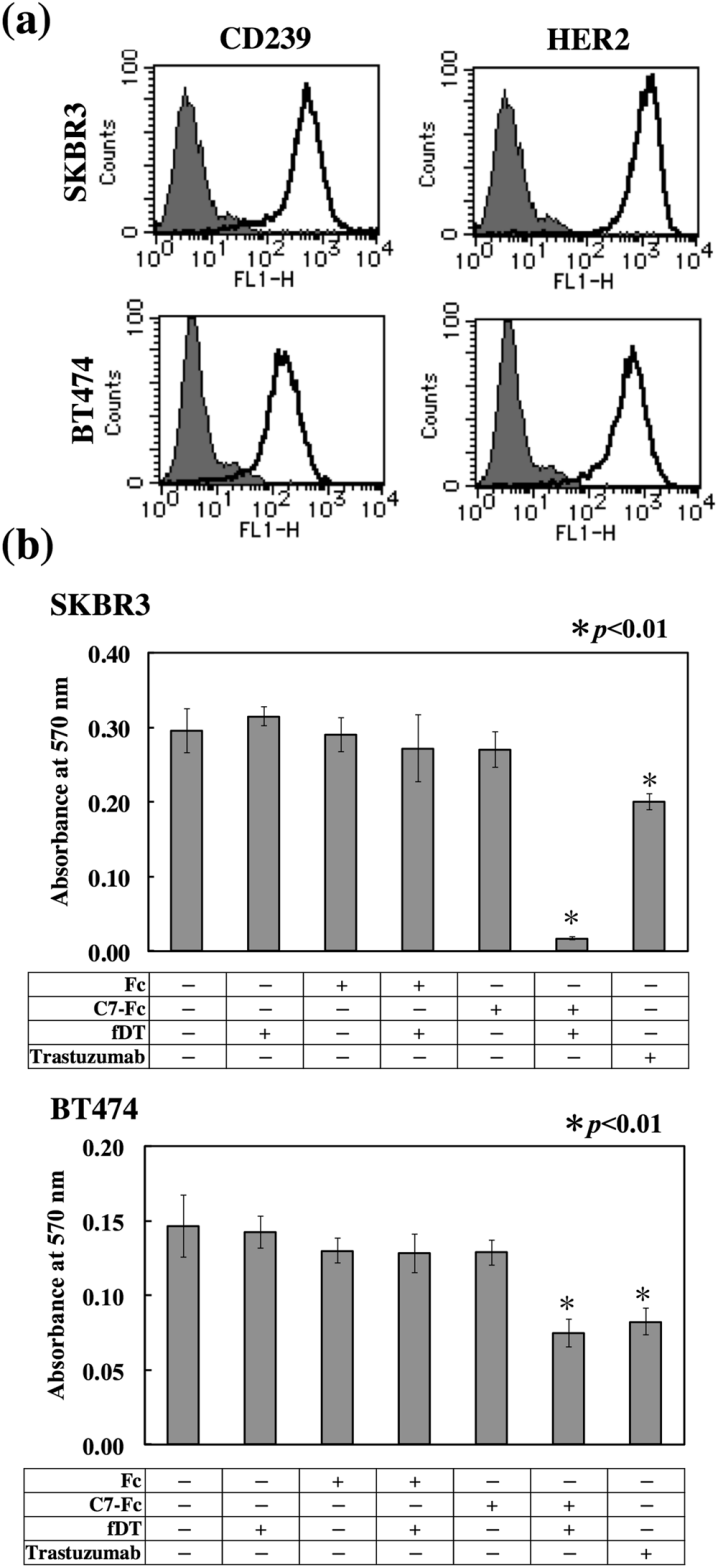
Cytotoxicity of fDT-bound recombinant antibody on CD239-highly positive breast cancer cells. (**a**) Flow cytometric analysis of CD239 and HER2 expression in SKBR3 and BT474 cells. Solid lines show C7-Fc and trastuzumab antibodies, and grey fill indicates the control Fc recombinant protein. (**b**) Cytotoxicity of fDT-bound C7-Fc. The recombinant proteins (C7-Fc and Fc) were combined with the fragment of diphtheria toxin (fDT). SKBR3 and BT474 cells were cultured in the presence of the recombinant proteins or fDT-bound recombinant proteins. After 4 days in culture, the number of cells was determined.

**Figure 5:**
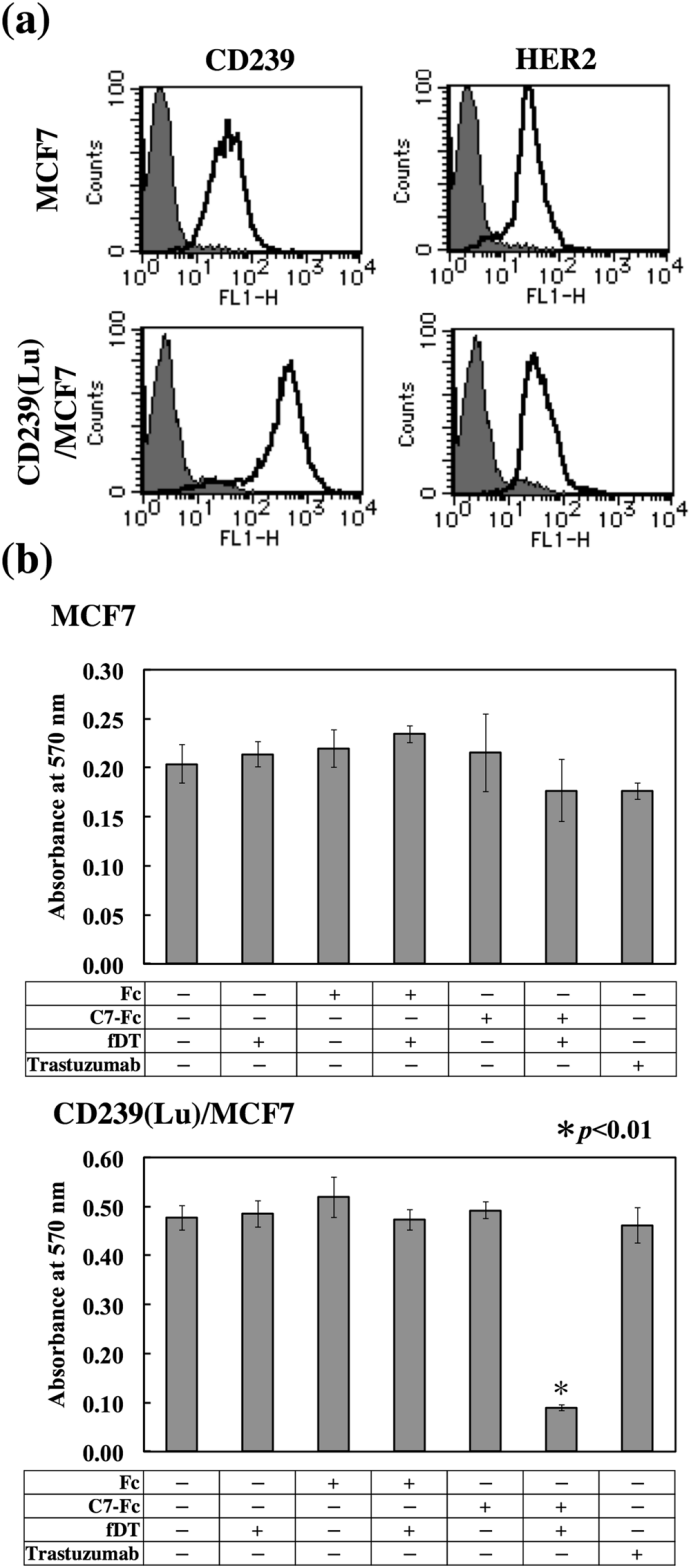
Cytotoxicity of fDT-bound C7-Fc antibody in breast cancer cells with different levels of CD239 expression. (**a**) Flow cytometric analysis of CD239 expression in MCF7 cells (left panels) and transfectants (right panels). Solid lines show C7-Fc (CD239, upper panel) and Herceptin (HER2, lower panel); grey fill indicates Fc recombinant protein and control antibody. CD239 was highly expressed on transfectants. (**b**) Cytotoxicity of fDT-bound C7-Fc in parent cells (MCF7) and transfectants (CD239(Lu)MCF7). Cell proliferation was measured as described above. fDT-bound C7-Fc effectively killed CD239-overexpressing cells.

We also examined the anticancer activity of fDT-bound C7-Fc in breast cancer cell lines that highly express CD239 on their cell surfaces (Fig. 4a). Similar to the mouse mAbs, although C7-Fc alone was not effective at killing SKBR3 or BT474 cells, fDT-bound C7-Fc exhibited cytotoxicity in both lines (Fig. 4b). The cytotoxicity of fDT-bound C7-Fc on BT474 cells was less effective than on SKBR3 cells, seemingly due the expression of CD239 in BT474 being lower than in SKBR3. We also observed that fDT-bound C7-Fc was not effective on CD239-moderately positive MCF7 cells (Fig. 5). Similarly, although trastuzumab significantly suppressed the proliferation of the HER2-highly positive SKBR3 and BT474 cells, it was not effective on the HER2-moderately positive MCF7 cells. These results suggest that the cytotoxicity of fDT-bound C7-Fc is dependent on the expression level of CD239. To investigate this hypothesis, we produced MCF7 cells overexpressing CD239(Lu) (Fig. 5a). CD239(Lu)-overexpressing MCF7 cells were sensitive to fDT-bound C7-Fc, indicating that its cytotoxicity requires high expression levels of CD239 on the cell surface (Fig. 5b).

### Cytotoxicity of fDT-bound C7-Fc on normal cells and transfectants expressing CD239 isoforms or a fusion protein

**Figure 6:**
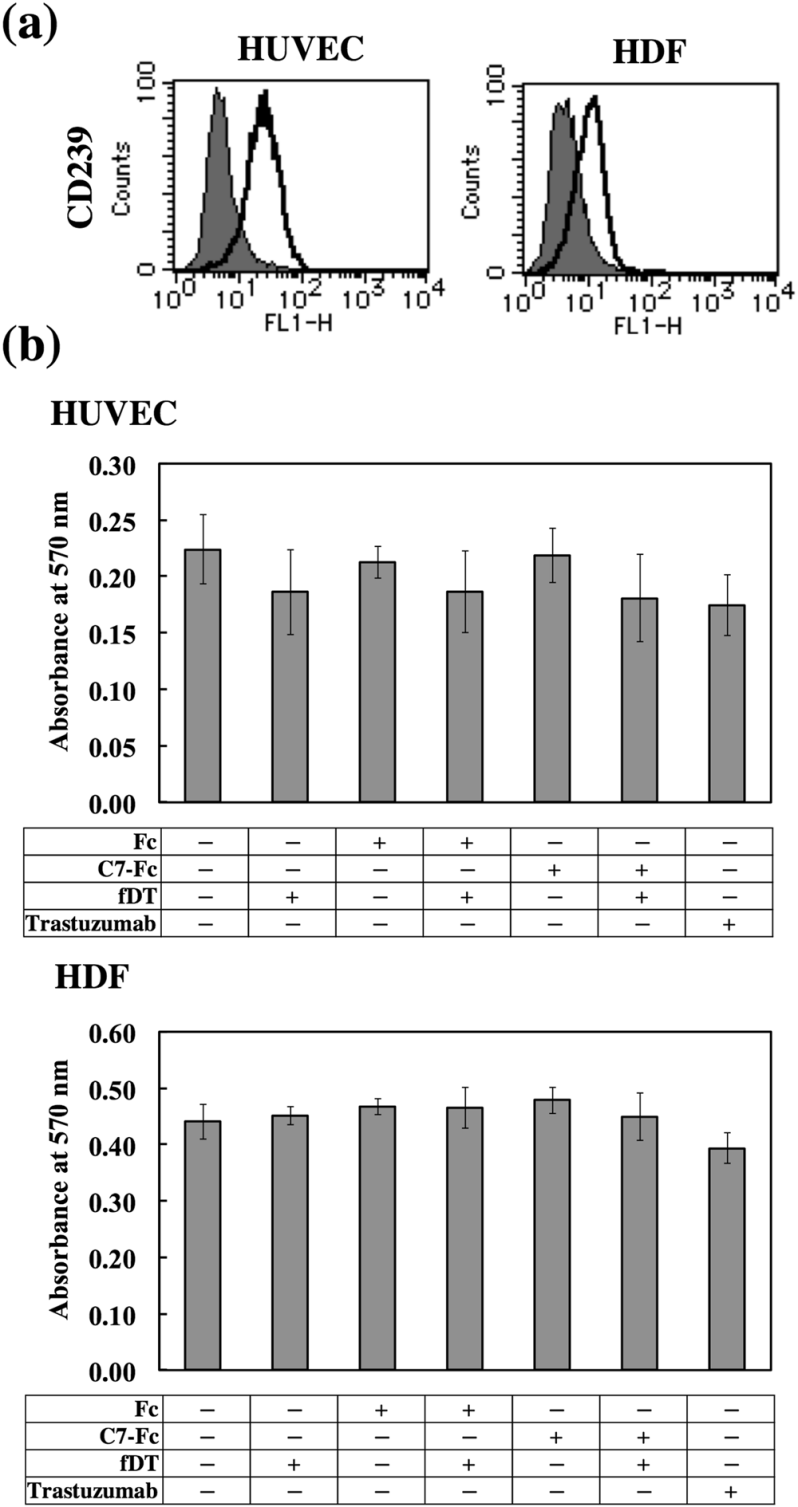
Lack of cytotoxicity of fDT-bound C7-Fc on normal human cells. (**a**) Flow cytometric analysis of CD239 expression in HUVECs (left panel) and HDFs (right panel). Solid lines show C7-Fc (CD239), and grey fill indicates the control Fc recombinant protein. Both normal cell types expressed CD239 weakly at the cell surface. (**b**) Cytotoxicity of fDT-bound C7-Fc in HUVECs and HDFs. The recombinant proteins (C7-Fc and Fc) were combined with the fragment of diphtheria toxin (fDT). HDFs and HUVECs were cultured in the presence of C7-Fc or fDT-bound C7-Fc. After 4 days in culture, the number of cells was determined. No cytotoxicity of fDT-bound C7-Fc on the normal cells was observed.

**Figure 7:**
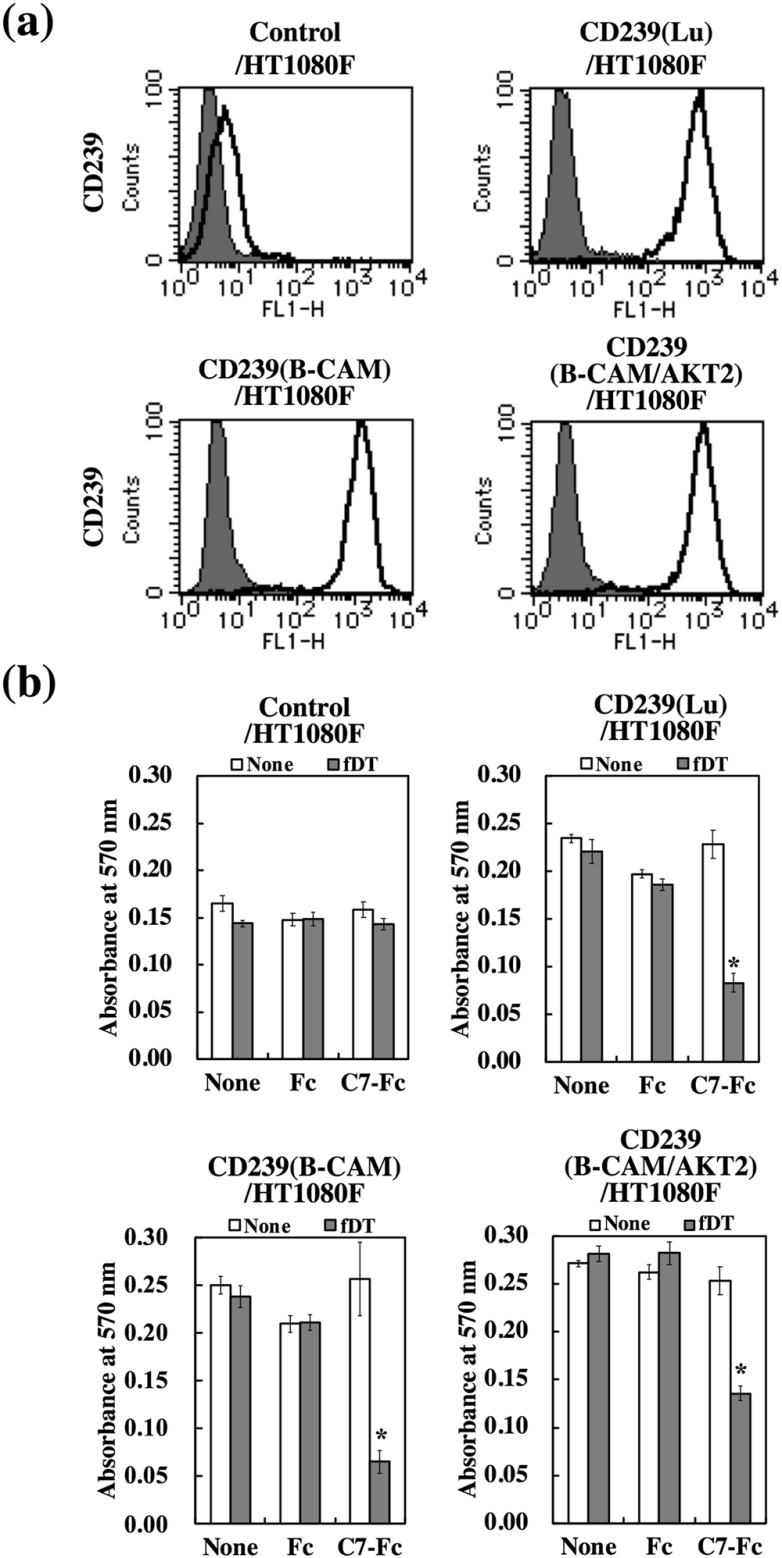
Cytotoxicity of fDT-bound C7-Fc on transfectants expressing CD239 isoforms or a fusion protein. (**a**) Flow cytometric analysis of transfectants expressing CD239 isoforms (Lu and B-CAM) or a fusion protein (B-CAM/AKT2). Solid lines show the C7-Fc antibody, and grey fill indicates the control Fc recombinant protein. (**b**) Cytotoxicity of fDT-bound C7-Fc in transfectants expressing CD239 isoforms (CD239(Lu)^+^ HT1080F and CD239(B-CAM) HT1080F) and a fusion protein (CD239(B-CAM/AKT2)HT1080F). Control cells (Control/HT1080F) and transfectants were cultured in the presence of C7-Fc or fDT-bound C7-Fc. After 4 days in culture, the number of cells was determined. *P < 0.01 by *t*-test.

Targeted anticancer drugs are necessary to minimize cytotoxicity for normal cells. We examined the cytotoxicity of fDT-bound C7-Fc for human umbilical endothelial cells (HUVECs) and dermal fibroblasts (HDFs). As shown in Fig. 6a, both normal cell types weakly expressed CD239 on their surface. We further observed that fDT-bound C7-Fc was not cytotoxic to HUVECs and HDFs (Fig. 6b). We examined if there was differential sensitivity for fDT-bound C7-Fc using transfectants expressing the CD239 isoforms Lu and B-CAM. The transfectants highly expressed Lu and B-CAM at comparable levels (Fig. 7a). Proliferation of both transfectants was similarly inhibited in the presence of fDT-bound C7-Fc (Fig. 7b). Recently, Kannan *et al*. reported a cancer-specific gene fusion between B-CAM and AKT2^19^. AKT2 is a key kinase in the PI3K signalling pathway that regulates cellular metabolism, proliferation, and migration^20^. Although its functions are unclear, the fusion protein is a candidate for targeted ovarian cancer therapy. To examine whether B-CAM/AKT2 was internalized into the cells, transfectants expressing the chimeric protein were generated. A growth assay showed that CD239- and B-CAM/AKT2-expressing HT1080F cells were sensitive to fDT-bound C7-Fc. These results indicate that the internalization of CD239 is not dependent on the cytoplasmic domain.

## Discussion

To explore whether CD239 could serve as a target antigen for diagnosis and therapy of breast cancer, we (a) immunohistochemically analysed CD239 expression in breast cancer tissues; (b) examined internalization of CD239 into malignant breast cancer cells *in vitro*; (c) produced a human scFv-Fc antibody (C7-Fc) against CD239; and (d) investigated the efficacy of C7-Fc as the basis for a novel ADC *in vitro*. ADCs can be divided into three general components: the antibody, the linker, and the cytotoxic drug. The efficacy of ADCs is dictated in part by the differential expression of the target antigen in cancer versus normal tissue. In this study, we showed that CD239 serves as target antigen for ADCs. Several previous reports have shown that the expression of CD239 is up-regulated in tissues of cancers such as ovary, skin, and liver cancers^15,17,21^. We also observed the up-regulated expression of CD239 in twenty-five of thirty-four breast cancer tissues. The tissues were simultaneously stained with anti-HER2 mAb. HER2 is well known as a diagnostic marker for malignant breast cancer^2^. The expression of HER2 is usually accompanied by that of CD239. Our results suggest that CD239 is a secondary target for HER2-positive breast cancer. Furthermore, CD239 is also detected in HER2-negative breast cancer. The results suggest that CD239 is not only a useful marker for classifying malignant breast cancer, but also a candidate molecule for targeted therapy.

The feasibility of ADCs requires the internalization of antigen upon antibody binding^22^. Here, we showed that the antibodies bound to CD239 were internalized into breast cancer cells. The binding of ligands often triggers the internalization of cell surface proteins^23^. Because the internalization of CD239 was not observed in CD239-weakly positive cells, the binding of the antibody does not seem to stimulate endocytosis of CD239. The internalization of CD239 may be caused by the response to maintain the homeostatic expression level at the cell surface. CD239 belongs to a subgroup of the Ig superfamily, among which CD166 is internalized into ovary and lung cancer cells through the clathrin-dependent pathway^24^. CD239 may also be internalized by a similar endocytic pathway. Fluorescently labelling the fDT-bound C7-Fc would enable elucidation of the mechanism of cellular uptake.

In this study, we used HEK293 cells to produce a human scFv-Fc antibody that binds to CD239. Because C7-Fc consists of an scFv derived from a human gene and human IgG_1_ Fc, it is almost a fully human antibody, except for a portion of the linker. The format of scFv is often inadequate for antigen binding in comparison with the whole antibody. Although the *K*_d_ value of C7-Fc is lower than that of mouse anti-CD239 mAbs, it seems to be sufficient for binding to CD239. Therapeutic antibodies possess multiple mechanisms for killing cancer cells. The antibody-dependent cell-mediated cytotoxicity (ADCC) is a mechanism of cell-mediated immune defence that results in target cell lysis^25^. Because there is a possibility that cancer patients have autoantibodies against CD239, the C7-Fc antibody may be able to stimulate ADCC.

The C7-Fc antibody was combined with a fragment of diphtheria toxin lacking the receptor-binding domain (fDT). fDT was a useful tool for evaluating the internalization of antibody bound to CD239. However, because fDT causes an immune response in humans, improvements are required before clinical use. As with trastuzumab emtansine (T-DM1), small molecules such as tubulin polymerization inhibitors, DNA alkylating agents, and enediyne antibiotics are ideal candidates for usage as toxic agents conjugated to the C7-Fc antibody. Furthermore, liposomes represent well-established vehicles for drug delivery. These include immunoliposomes, which consist of an antibody and a long-circulating liposome as a nanoparticle-based drug delivery system. Liposomes conjugated with a fragment of anti-HER2 antibody has been delivered to HER2-overexpressing cancer cells^26^. C7-Fc would be a useful antibody for generating immunoliposomes that can be specifically delivered to not only CD239-positive but also CD239-positive/HER2-negative breast cancer tissues.

In conclusion, CD239 serves as a target antigen associated with breast cancer. The antibodies binding to CD239 are internalized into cancer cells. This satisfies a requirement of the antibody to be used for ADCs. ADCs can be conjugated to highly toxic agents including radioisotopes and systemic toxins. Anti-CD239 antibody (C7-Fc) already consists of parts of human immunoglobulin. Therefore, the choice of toxic agents conjugated to C7-Fc will be important for creating useful anticancer drugs. CD239 and human anti-CD239 antibodies will provide opportunities for improving the diagnosis and therapy of breast cancers and other neoplasias.

## Methods

### Antibodies and regents

mAbs against CD239 were purchased from R&D Systems (87202 and 87207), Minneapolis, MN, and from Serotec (BRIC221), Oxford, UK. Anti-HER2/ERBB2 rabbit mAb (29D8) was purchased from Cell Signaling Technology, Danvers, MA. Biotinylated anti-human IgG_1_ Fc antibody was purchased from Jackson ImmunoResearch, West Grove, PA. Horseradish peroxidase-conjugated streptavidin and Alexa Fluor 488- and 594-conjugated secondary antibodies were purchased from ThermoFisher Scientific, Waltham, MA. Herceptin was purchased from Chugai Pharmaceutical, Tokyo, Japan. The fragment of diphtheria toxin fused with C1-3 domains of *Streptococcus* protein G was prepared as described previously^18^. Recombinant proteins containing the CD239 extracellular domain fused with a 6 × His-Tag (Sol-Lu) or Fc-Tag (Lu-Fc), and the control recombinant protein (Fc) were produced and characterized as described previously^11,27^.

### Immunohistochemistry

The tissue array was purchased from BioChain, Newark, CA. Acetone-fixed sections were blocked in 10% normal goat serum and then incubated with mouse anti-CD239 (87207) and rabbit anti-HER2 (29D8) mAbs. Both antibodies were diluted with Ca^2+^- and Mg^2+^-free phosphate-buffered saline (PBS(−)) containing 1% bovine serum albumin (BSA); all washes were performed in PBS(−). Secondary antibodies conjugated to Alexa Fluor 488 or 594 were used. After several washes, sections were mounted in 90% glycerol containing 0.1× PBS and 1 mg/mL *p*-phenylenediamine. Images were captured using a Biozero microscope (Keyence, Osaka, Japan). The scaling of HER2 expression was performed according to the guidelines^28^. The expression of CD239 was defined similarly. Strong (+++) is based on circumferential membrane staining that is complete and intense. Moderate (++) is defined by circumferential membrane staining that is incomplete and/or weak/moderate. Weak (+) is based on incomplete membrane staining that is faint. N.D. (−) is negative staining.

### Cell culture

HEK293, BT474, and SKBR3 cells were purchased from ATCC, Manassas, VA, and MCF7 cells from Health Science Research Resources Bank, Osaka, Japan. Human dermal fibroblasts (HDFs) were purchased from Cell Applications, San Diego, CA. HEK293, BT474, and MCF7 cells and HDFs were maintained in DMEM containing 10% FBS, while SKBR3 cells were maintained in McCoy’s 5 A medium containing 10% FBS. Human umbilical vein endothelial cells (HUVECs) were purchased from Lonza, Basel, Switzerland, and maintained in MCDB107 medium (Funakoshi, Tokyo, Japan) supplemented with 10% FBS, 20 ng/mL bovine brain extract (Funakoshi), 10 ng/mL EGF (BD Biosciences, Bedford, MA), and 50 μg/mL heparin (Wako, Osaka, Japan). For HUVECs, the culture dishes were coated with 0.3 mg/mL type I collagen (Koken, Tokyo) at 37 °C for 1 h. All cells were maintained at 37 °C in a humidified 5% CO_2_/95% air atmosphere.

### Flow cytometric analysis

Cells were removed using cell dissociation buffer (ThermoFisher Scientific) and suspended in PBS(−) containing 0.1% BSA and 1 mM EDTA. The suspended cells were incubated with anti-CD239 (87207 or C7-Fc) or -HER2 (Herceptin) mAb for 1 hour at 4 °C. After washing with PBS(−) containing 0.1% BSA and 1 mM EDTA, the cells were incubated with Alexa Fluor 488-labelled secondary antibody for 1 h at 4 °C. The cells were then analysed on a FACScalibur flow cytometer (Becton Dickinson, San Jose, CA).

### Cell growth assays

Cells were harvested with trypsin and adjusted at 40,000 cells/mL in growth medium containing 10% Ultra Low IgG FBS (ThermoFisher Scientific). The cell suspensions were placed into 96-well cell culture plates at 4,000 cells/well. After culturing for 1 day, the antibodies bound with or without fDT were added to the wells. After an additional 4 days of culture, the number of viable cells was determined using 3-(4,5-dimethylthiazol-2-yl)-2,5-diphenyltetrazolium bromide (MTT; Dojin, Kumamoto, Japan) reduction assay.

### Production of scFv-Fc antibody

DNA encoding C7 was purified from a phage clone displaying scFv against CD239^29^ and amplified using a primer set (Table S2). The PCR products were seamlessly joined to DNA encoding the human laminin γ2 signal sequence by sequential PCR, and restriction sites were introduced at appropriate locations. The PCR products were subcloned into a human IgG_1_ Fc expression vector. HEK293 cells were transfected with the C7-Fc expression vector using Lipofectamine 2000 (ThermoFisher Scientific). Transfectants were grown to confluency in culture dishes with DMEM containing 10% FBS and 100 μg/mL zeocin (ThermoFisher Scientific) and incubated in serum free DMEM for 4 days. The recombinant antibody was purified from the conditioned media by Protein A Sepharose (GE Healthcare, Little Chalfont, UK), as described^14^. The purified proteins were verified by SDS-PAGE using 12.5% gels under reducing conditions.

### Enzyme-linked immunosorbent assay (ELISA)

Ninety-six-well ELISA plates (ThermoFisher Scientific) were coated with recombinant CD239 (Sol-Lu) and blocked with BSA in PBS(−). C7-Fc was added and incubated at room temperature for 1 h. After washing with PBS-T, the bound C7-Fc was detected using a biotinylated anti-human IgG_1_ Fc mAb. After further washing, the bound antibodies were detected by addition of streptavidin-conjugated horseradish peroxidase, followed by addition of 0.4 mg/mL *o*-phenylenediamine and 0.01% H_2_O_2_. The absorbance was measured at 450 nm on a microplate reader (BIO-RAD, Hercules, CA).

### Surface plasmon resonance analysis

The binding affinities of C7-Fc and anti-CD239 mAbs were assayed by surface plasmon resonance analysis using a Biacore T200 system (GE Healthcare Bio-Sciences). The recombinant CD239 (Lu-Fc) was immobilized on a CM5 sensor chip (GE Healthcare Bio-Sciences). Various amounts of C7-Fc and anti-CD239 mAbs in 10 mM HEPES (pH 7.4), containing 150 mM NaCl, 3 mM EDTA, and 0.005% Tween 20, were injected into the CD239-immobilized flow cell with a flow rate of 50 μL/min. The *K*_*d*_ was determined by a nonlinear fitting method using the BIAevaluation software (GE Healthcare Bio-Sciences).

### Construction of expression vectors

The DNA encoding CD239 (Lu) was amplified using a primer set listed in Table S2, as described in our previous study^11^. The DNA fragment was inserted into pIRESpuro3 (Takara Bio Inc., Tokyo, Japan). For expression of the B-CAM/AKT2 fusion protein, full-length cDNA for human AKT2^30,31^ was obtained from RIKEN BRC (Tsukuba, Japan) and used as a template for PCR. DNA encoding the fusion protein was generated by PCR and inserted into pcDNA5/FRT for stable high-level expression *via* the Flp-In System (ThermoFisher Scientific).

### Generation of stable expression cell lines

MCF7 cells were transfected with the human CD239 (Lu) expression vector using Lipofectamine 2000 (ThermoFisher Scientific). CD239-highly positive MCF7 cells were cloned and cultured in the presence of 0.5 μg/mL puromycin (ThermoFisher Scientific). The Flp-In system (ThermoFisher Scientific) was used for the generation of a stable B-CAM/AKT2 expressing cell line. HT1080F cells carrying the FRT-site sequence were co-transfected with the fusion protein expression vector and the FLP-recombinase vector (pOG44). Stable cell lines expressing B-CAM/AKT2 were selected using 100 μg/mL hygromycin (ThermoFisher Scientific).

### Statistical Analysis

Each bar represents the mean of triplicate assays. Error bars indicate standard deviation. Statistical significance was determined using Student’s *t* test.

## Electronic supplementary material

Supplementary information
