# Supplementary material for: Internalization of CD239 highly expressed in breast cancer cells: a potential antigen for antibody-drug conjugates
Source: Sci Rep. 2018 Apr 26;8:6612. doi: 10.1038/s41598-018-24961-4 (PMC5919910; doi:10.1038/s41598-018-24961-4)
Supplement: Supplementary file 1 — Supplementary information [file 41598_2018_24961_MOESM1_ESM.pdf]

Supplementary information

Internalization of CD239 highly expressed in breast cancer cells: a potential antigen for antibody-drug conjugates

Yamato Kikkawa[1*], Yurie Enomoto-Okawa[4], Aiko Fujiyama[4], Takeshi Fukuhara[2#], Nozomi Harashima[1], Yumika Sugawara[1], Yoichi Negishi[3], Fumihiko Katagiri[1], Kentaro Hozumi[1], Motoyoshi Nomizu[1], and Yuji Ito[4]

[1]Department of Clinical Biochemistry and [2]Laboratory of Oncology, [3]Department of Drug Delivery and Molecular Biopharmaceutics, Tokyo University of Pharmacy and Life Sciences, Tokyo 192-0392, Japan; [4]Graduate School of Science and Engineering, Kagoshima University, Kagoshima 890-0065, Japan

#Present address:
Department of Neurology, Graduate School of Medicine, Juntendo University, Bunkyo-ku, Tokyo, 113−8421, Japan

*Corresponding author

Table 1S Summary of CD239 and HER2 expression in the breast cancer tissues				
Patient No.	Pathological Diagnosis	Age	CD239	HER2
1	Invasive ductal carcinoma	64	++	++
2	Invasive ductal carcinoma	70	+	-
3	Invasive ductal carcinoma	48	+	++
4	Invasive ductal carcinoma	57	+	+
5	Invasive ductal carcinoma	65	-	-
6	Invasive ductal carcinoma	65	-	-
7	Invasive ductal carcinoma	82	++	-
8	Invasive ductal carcinoma	34	+++	-
9	Invasive ductal carcinoma	48	+++	+++
10	Invasive ductal carcinoma	57	+	+
11	Invasive ductal carcinoma	77	+	-
12	Invasive ductal carcinoma	42	+++	-
13	Invasive ductal carcinoma	47	++	+++
14	Invasive ductal carcinoma	34	+	-
15	Invasive ductal carcinoma	48	+	+++
16	Invasive ductal carcinoma	41	+	-
17	Invasive ductal carcinoma	47	-	-
18	Invasive ductal carcinoma	37	-	-
19	Invasive ductal carcinoma	32	+	-
20	Invasive ductal carcinoma	63	-	-
21	Invasive ductal carcinoma	50	+	+
22	Invasive ductal carcinoma	56	+++	+++
23	Invasive ductal carcinoma	56	+	+++
24	Invasive ductal carcinoma	41	+++	+++
25	Invasive ductal carcinoma	47	++	++
26	Invasive ductal carcinoma	60	++	-
27	Invasive ductal carcinoma	46	+++	-
28	Invasive ductal carcinoma	45	-	-
29	Invasive ductal carcinoma	57	+	-
30	Invasive ductal carcinoma	37	-	-
31	Invasive ductal carcinoma	57	-	-
32	Invasive ductal carcinoma	67	-	-
33	Invasive ductal carcinoma	63	+	-
34	Invasive ductal carcinoma	50	++	+++

Table 2S Primer sets		
Protein	Primer	Sequence (5'-3')
Signal sequence of human laminin γ2 chain	HG2-03	CGCAATTGACCGCCATGCCTGCGCTCTG
	HG2-04	AGACTGCACCAGCTGCATCTGCACTTCCCTCCTGGAGGTGGC
C7 scFv	C7-01	GCCACCTCCAGGAGGGAAGTGCAGTAGCAGCTGGTGCAGTCT
	C7-02	CGCCTAGGCCTAGGACGGTCAGCTTGGTCCC
Human CD239(Lu)	HLU03	GGAATTCGCCACCATGGAGCCCCCGGACGCACCG
	HLU20	CGGGATCCTCAGCACTCGTCTCCGAAGCCCCC
Fusion protein of B-CAM and AKT2	HLU121	GCACTGCTAGCACCGCCATGGAGCCCCCGGACGCACCG
	HLU168	CTGGATGGCCCGCATCCACTCCTCCGGAGCCCCCTTCTCCCGCCGCTG
	AKT2_01	CAGCGGCGGGAGAAGGGGGCTCCGGAGGAGTGGATGCGGGCCATCCAG
	AKT2_02	CGTGAAAGCTTCAGGTACCCTCGCGGATGCTGGCCGAGTAGGA
